# Safety and tolerability of virucidal hand rubs: a randomized, double-blind, cross-over trial

**DOI:** 10.1186/1753-6561-5-S6-P271

**Published:** 2011-06-29

**Authors:** A Conrad, D Cosic, C Schmoor, M Dettenkofer

**Affiliations:** 1 Department of Environmental Health Sciences, University Medical Center Freiburg, Freiburg, Germany; 2 Clinical Trials Unit, University Medical Center Freiburg, Freiburg, Germany

## Introduction / objectives

Clinical trial to evaluate the safety and tolerability of different virucidal hand rubs.

## Methods

In a randomized, double-blind, 4-period cross-over trial, healthy volunteers received 3 different virucidal hand rubs (P1-3) and a reference product (R) in a randomized sequence each over a period of 4 days (90 mL/d) with a wash-out period of 10 days. Primary endpoint was skin barrier function measured by transepidermal water loss (TEWL) in g/hm^2^ at the end of the 4-day application period. Secondary endpoints were corneometry, skin status score, and adverse events (AE). TEWL and corneometry were analyzed in linear models. The effects were estimated as differences between agents with 95%CI and tested with a 2-sided level of 0.05.

## Results

22 subjects (7 males, 15 females) were randomized and started at least one period. TEWL was 22.5, 95%>CI [19.6,25.4], 16.3 [13.5,19.1], 16.4 [13.4,19.3], and 24.0 [21.1,27.0] after P1, P2, P3, and R; p<0.0001. Corneometry was 49.7 [40.7,58.6], 45.4 [36.9,53.9], 64.3 [55.3,73.2], and 48.9 [39.6,58.1] after P1, P2, P3, R; p=0.005. The median skin status score dorsal was 0.5, 0, 1, 0.5 after P1, P2, P3, R. The percentage of subjects who experienced at least one AE was 86%, 25%, 89%, and 56% with P1, P2, P3, R. The majority of AEs were skin reactions (no serious AEs).

## Conclusion

Results were inconsistent, e.g. P3 showed low TEWL (= fair skin barrier function) but high skin status score and high number of adverse events (= impaired tolerability). Assessment of TEWL may be influenced by non volatile compounds in this setting and not be a suitable measure. Number of AEs was higher in products containing phosphoric acid (P1, P3). In general, number of AEs was higher than expected for all products requiring further improvement in hand rub development.

## Disclosure of interest

A. Conrad Grant/Research support from B.Braun, Melsungen, Germany, D. Cosic: None declared, C. Schmoor: None declared, M. Dettenkofer Grant/Research support from B.Braun, Melsungen, Germany.

